# Meta-analysis reveals severe pollen limitation for the flowering plants growing in East Himalaya-Hengduan Mountains region

**DOI:** 10.1186/s12898-020-00322-6

**Published:** 2020-09-29

**Authors:** Xianfeng Jiang, Yanping Xie

**Affiliations:** 1 College of Agriculture and Bioscience, Dali University, Dali, 671003 Yunnan China; 2 College of Life Sciences, Huaibei Normal University, Huaibei, 235000 Anhui China

**Keywords:** Pollen limitation, Flowering plants, Meta-analysis, Eastern himalaya, Hengduan mountain

## Abstract

**Background:**

Pollen limitation occurs widely and has an important effect on flowering plants. The East Himalaya-Hengduan Mountains region is a global biodiversity hotspot. However, to our knowledge, no study has synthetically assessed the degree of pollen limitation in this area. The present study aims to reveal the degree of pollen limitation for the flowering plants growing on East Himalaya-Hengduan Mountains and to test whether the reproductive features or the elevation is closely correlated with the degree of pollen limitation in this area.

**Results:**

We complied data from 76 studies, which included 96 species and 108 independent data records. We found that the flowering plants in this area undergo severe pollen limitation [overall Hedges’ d = 2.004, with a 95% confidence interval (1.3264, 2.6743)] that is much higher than that of the flowering plants growing in many other regions around the world. The degree of pollen limitation was tested to determine the correlation with the capacity for autonomous self-reproduction and with the pollination pattern (generalized vs. specialized pollination) of plants. In addition, we found a clear relationship between elevation and the degree of pollen limitation, which indicates that plants might undergo more severe pollen limitation in relatively high places.

**Conclusions:**

This paper is the first to address the severe pollen limitation of the flowering plants growing in East Himalaya-Hengduan Mountains region. Moreover, we reveal the positive correlation between elevation and the degree of pollen limitation.

## Background

Pollen limitation widely occurs and has been an essential clue for determining whether flowering plants undergo limitation from pollination services in natural habitats [1, 2]. Flowering plants, especially those obligately cross-fertilized, strongly rely on external pollination services for their sexual reproduction [3, 4]. On one hand, pollen limitation exerts an essential selection on plants [5–7]. Within a life-history cycle, severe pollen limitation during a flowering season would cause the failure of sexual reproduction in a given year, which might therefore cause reproductive resources discounting for plants [8]. Over the long term, plants might evolve favorable strategies that ensure their reproductive success, i.e., autonomous self-reproduction, clonal growth or apomixis, if they undergo long-term pollen limitation [9]. On the other hand, the pollination services in nature are largely provided by pollinators (bees, butterflies, birds, etc.) [4]; thus, the degree of pollen limitation could indicate the abundance and variability of the pollinators in nature to some extent.

Various independent studies have reported that many plant species do suffer from pollen limitations in nature. Especially those plants grow in natural habitats, for example, *Primula modesta*, an obligate heterostylous outcrossing plant that is not pollination limited in domestic environment, but suffers severe pollen limitation in the field [10]. In general, plants growing in harsh environments, i.e., deserts, arctic and alpine areas, are more frequently pollen limited [11, 12]. That is probably because the abundance and vitality of pollination insects in these areas are generally lower than those of pollination insects in tropical and subtropical areas. Several synthetic reviews have assessed the frequency and degree to which pollen limitation occurs in different areas around the world [13]. For example, pollen limitation has been studied among alpine flowering plants [14] and plants of the Atlantic forest in Brazil [15].

**Fig. 1:**
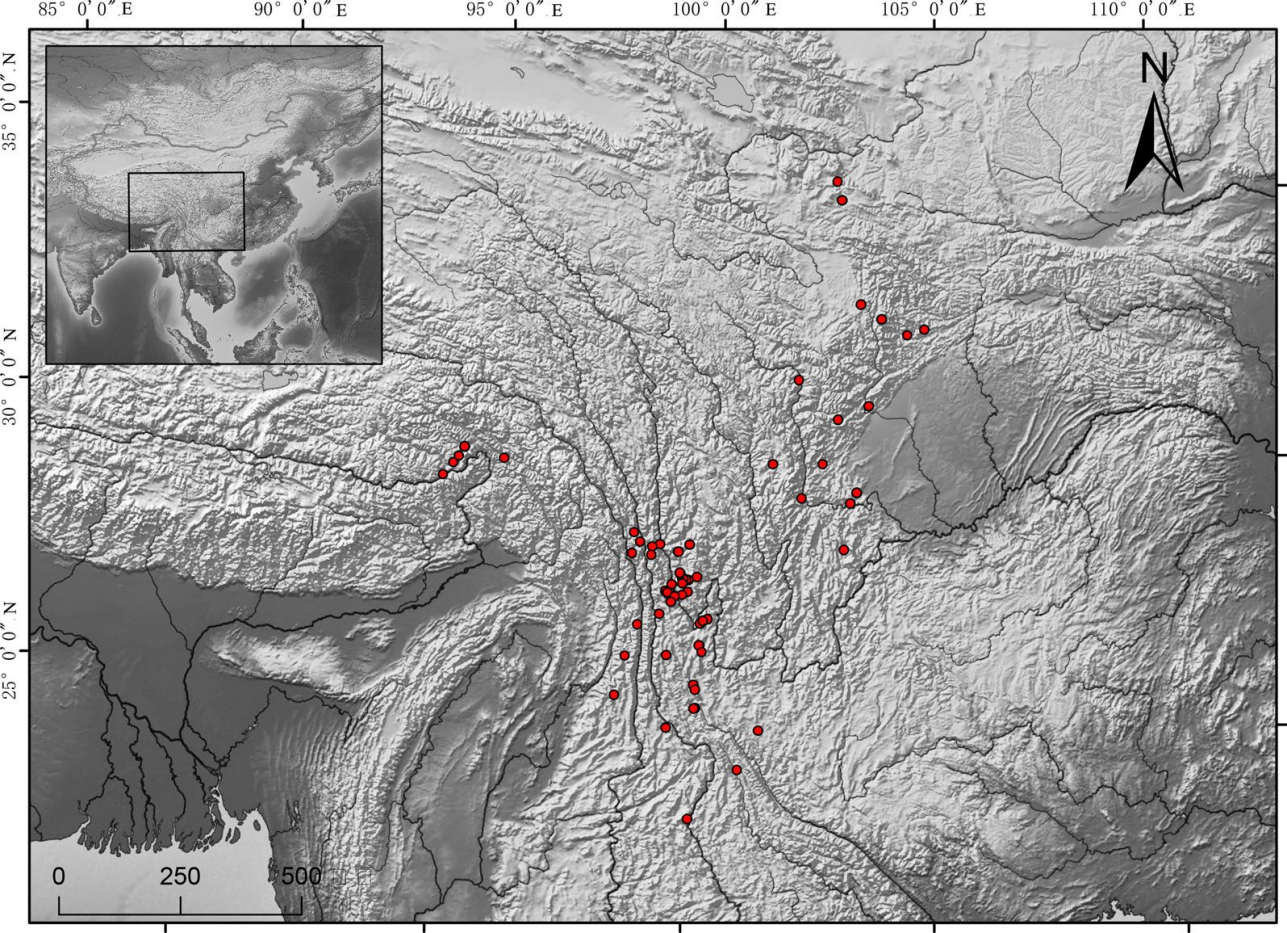
The locations of the plant species collected in this synthetic analysis. All the plant species are distributed within the East Himalaya-Hengduan Mountains region (including northwest of Yunnan province, west of Sichuan province, southeast Tibet and south of Gansu province). The map is from Natural Earth, which was built through a collaboration of many volunteers and is supported by NACIS (North American Cartographic Information Society), and is free for use in any type of project, https://www.naturalearthdata.com/

East Himalaya-Hengduan Mountains region locates in southwestern China and includes northwestern Yunnan Province, western Sichuan province and southeastern Tibet (Fig. 1). In Chinese, “Hengduan” means lands separated by mountains and rivers transversely. Mekong River, Yangzi River and Salween River, along with many different mountains, go across this area from north to south, dividing this region into pieces. The East Himalaya-Hengduan Mountains region varies in elevation from 1200 m to more than 6000 m. Benefit from its geography feature, this area contains various climates and vegetation types, from tropical seasonal forests to subtropical forests and temperate meadows. Meanwhile, many plant species get differentiated in this region during Quaternary glacial and interglacial periods [16, 17]. For instance, the East Himalaya-Hengduan Mountains region is the biodiversity and evolutionary center of the families Primulaceae [18], Ericaceae and Gentianaceae [19].

Previous synthetic studies on the degree of pollen limitation in other places around the world generally focused on various flower traits such as the capacity for autonomous self-reproduction, the vegetation type and the flower shape [14, 15]. In the present study, the capacity of autonomous selfing, the pollination pattern, the flower shape and the reward type are included to the analysis. Meanwhile, given that the abundance and vitality of pollinators are commonly regarded to decrease with increasing elevation [20], elevation is probably an important factor affecting the degree of pollen limitation [21]. Even so, some studies also provide evidence that elevation could affect the pollinator abundance but hardly on the reproductive success of plants [22]. Considering that the elevation of the East Himalaya-Hengduan Mountains region varies substantially, this area is an ideal model to study the relationship between elevation and the degree of pollen limitation. In order to synthetically assess the relationship between elevation and the degree of pollen limitation, we include elevation as a continuous explanatory factor in our analysis as well.

In the present study, we aim to answer the following specific scientific questions: (1) What is the degree of pollen limitation for the flowering plants growing on the East Himalaya-Hengduan Mountains? (2) Which plant reproductive features significantly affect the degree of pollen limitation that a plant suffers? (3) Does the degree of pollen limitation show an obvious correlation with elevation in the East Himalaya-Hengduan Mountains region?

## Method

### Literature review and dataset

A search for the focal publications was primarily conducted in the Web of Science database (webofknowledge.com) and Google Scholar (scholar.google.com) using the keywords “Yunnan” or “Tibet” or “Sichuan” or “Himalaya” or “Hengduan” in combination with the terms “pollination” or “fruit set” or “seed set” or “hand poll*” or “supp* poll*”. To include more published pollination works conducted in this area, we also searched the Chinese publications database, CNKI (Chinese National Knowledge Infrastructure, www.cnki.net), using the keyword combinations mentioned above in Chinese. Some unpublished papers, theses and personal data graciously given by the authors were also added to this analysis. The following criteria were used to select the focal publications: (1) studies of flowering plants growing on the East Himalaya-Hengduan Mountains, including plants in northwestern Yunnan Province, western Sichuan Province, southern Gansu Province and southeastern Tibet; (2) studies that applied hand pollination on the studied plants and assed the reproductive success of both hand- and open-pollination treatments; and (3) studies whose results included average seed set/seed production, standard deviation and sample size and fruiting proportion and sample size. For the fruit data recorded, we extracted the numbers of flowers with and without fruit in both hand- and open-pollinated treatments. For the seed set or seed production data, we extracted the mean, standard deviation and sample size of both treatments. For those data exhibited in graphs, we extracted the dataset from the graphs with ImageJ 1.31 [23].

To understand the effects of plant reproductive features on the degree of pollen limitation, several key traits were extracted, including the capacity for autonomous self-reproduction, pollination type (generalized vs. specialized pollination) [24], flower shape (restricted vs. nonrestricted) [25], rewards type (nectariferous vs. nectarless) [26] and flowering time [27], from the collected studies, other studies on the same species or the Flora of China (www.iplant.cn). We assessed the capacity for autonomous self-reproduction by the reproductive success of bagged treatments and hand-pollination treatments from the collected papers or from other studies on the same species. The flowering time was separated into three categories: early spring (before May), summer (May to August), and autumn (August). The flowering time was separated into such way mostly based on the abundance of pollinators in different seasons in this region. The pollination pattern was assessed by the number of pollination functional groups according to the description in the paper. Specialized pollination plants had only one kind of pollination functional group (moth, bees, butterflies, ants, etc.); otherwise, a plant was regarded as having generalized pollination. The flower shape was separated into two types, one is the open flowers those were radial symmetry and whose nectars were easily to be reached (non-restricted flowers), and the other one was bilateral symmetry or those with a tubular shape that can limit pollinator’s behavior (restricted flowers). The rewards of a plant were also collected and separated into two categories, nectariferous vs. nectariless, based on the description in the paper or from the Flora of China. We also extracted the elevation from the publications if the authors provided it; otherwise, we extracted it from Google Maps based on the geographic coordinates or names the authors provided. The elevation was arranged as a continuous variable from 1213 to 4600 m.

Given that studies of heteromorphic species had more than one data entry, such as *Primula chungensis*, which has three different morphs (long-, short- and homo-styled morph), we took the average of records as the final number for a species if the data did not differ significantly in the capacity for autonomous selfing, elevation, pollination type, flowering time or rewards type. In total, 108 datasets from 94 species and 26 families were collected from 76 studies, and Primulaceae (14.04%), Gentianaceae (8.77%), Scrophulariaceae (10.53%) and Orchidaceae (14.91%) represented most of the collected species in our study.

### Data analysis

For the binary data, i.e., fruit set, we calculated the effect size with log odds ratios (ln(o)) obtained from a 2 X 2 table of the flowers with and without fruit in the hand- and open-pollinated treatments [28]. For the continuous data, i.e., seed set and seed production, we calculated the effect size by Hedges’ d with the mean, standard deviation and sample size of the hand- and open-pollinated treatments [28]. The standard error recorded in studies was transformed to standard deviation by dividing by the sqrt of the sample size. The effect size of each study was calculated in OpenMEE, a free meta-analysis software for ecology and evolution research [29]. To include as many datasets as possible, we transformed the log odds ratios to Hedges’ d following Coopers’ method [28]. The degree of pollen limitation was viewed as the reproductive success of hand-pollination treatment vs. open-pollination treatment, and the plant species were viewed as pollination limited if the effect size was positive and its 95% confidence interval did not overlap zero [30]; otherwise, it was not viewed as being pollination limited.

The overall effect size was calculated by the traditional method and phyloMeta method separately. For the traditional meta-analysis, we used a random model to calculate the overall effect size, which took into account the deviation from the true effect size that may be generated by differences between the studies. We calculated Rosenthal’s fail-safe numbers to test the presence of publication bias in the datasets [31]. These numbers represent the number of nonsignificant, unpublished, or missing studies that would need to be added to a meta-analysis to change the results from significant to nonsignificant. If the fail-safe number is larger than five times the sample size plus 10, it is safe to conclude that the results are robust with the consideration of publication bias [31]. Because the Rosenberg fail-safe number was 80,721 in the present study, which was much larger than the critical value (1080), there was no evidence of publication bias in the dataset. The classical meta-analysis, as well as the calculation of Rosenberg fail-safe number, was conducted with the “metaphor” package [32] in R 3.6.0.

For the phylogenetic meta-analysis, we constructed a list of plant species (family/genus/species) with the aid of the “plantlist” package [33]. The angiosperm APG III consensus tree was constructed [34], and the branch lengths were calibrated from the “ape” package [35], which is used for phylogenetic analysis. After that, the overall effect size of the phylogenetic meta-analysis was tested by the “metafor” package [32] with the aid of the constructed phylogenetic tree mentioned above. Since the heterogeneity of the dataset was significant, several explanatory variants were added in the analysis. In the analysis, the flowering time, the capacity of autonomous selfing, the pollination type, the flower shape and the reward type were set as discontinuous explanatory variables, and the elevation was set as a continuous explanatory variable. The Q_m_ and p values were calculated for each explanatory variable, and the different categories within an explanatory variable were significantly different if the p value did not exceed 0.05. The correlation between the continuous explanatory variable (elevation) and the degree of pollen limitation was also calculated. We also applied multiple analysis to test for potential interaction among different modulators.

## Results

### The degree of pollen limitation of the flowering plants in the East Himalaya-Hengduan Mountains

**Fig. 2:**
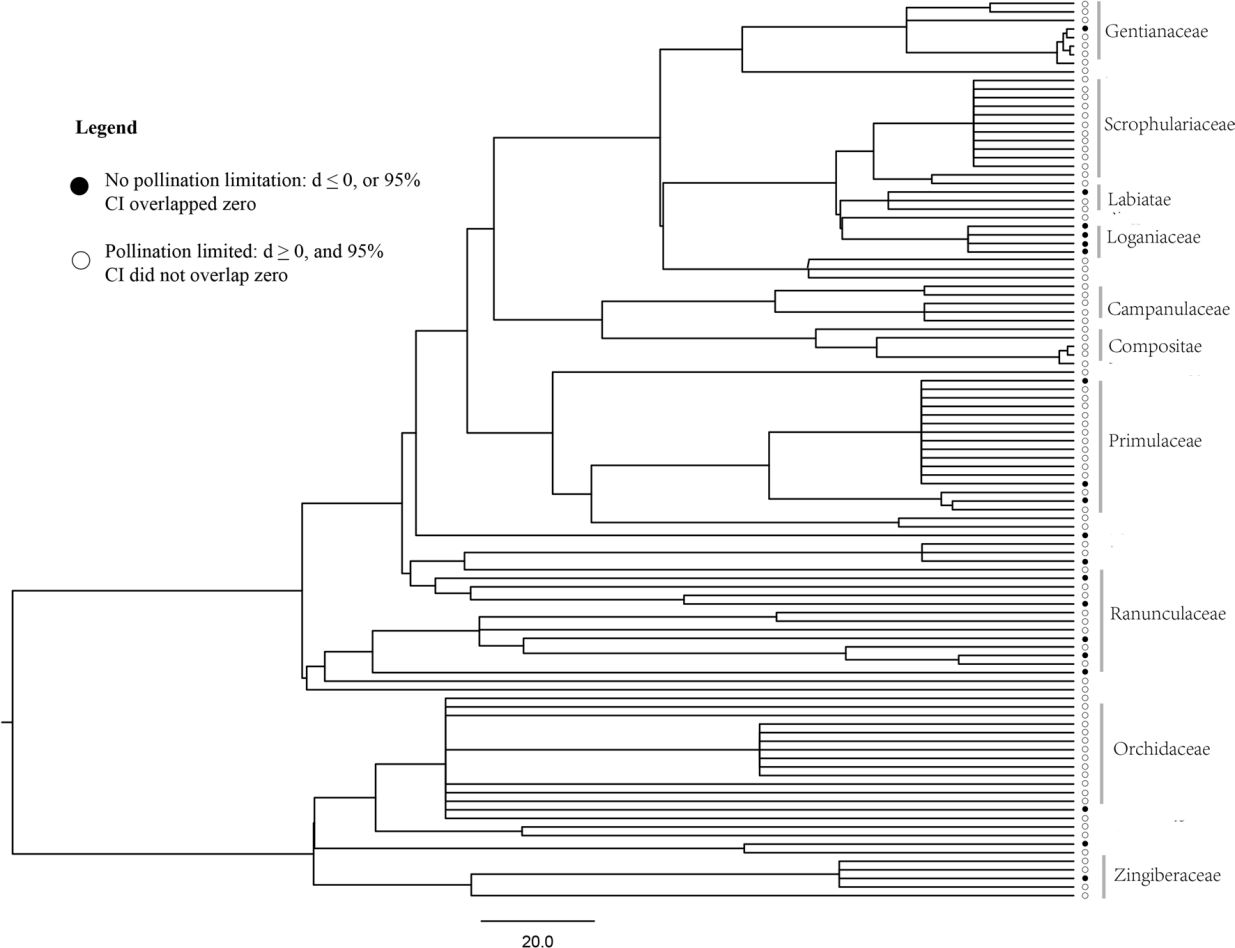
Distribution of pollination limitation of collected plants in the East Himalaya-Hengduan Mountains. The black circles represent the species that were not pollination limited (the effect size is negative or the 95% confidence interval overlap zero), and the white circles represent pollination-limited species (the effect size is positive and the 95% confidence interval did not overlap zero)

The overall effect size inferred by the traditional meta-analysis was 2.0004, with a 95% confidence interval [1.3264, 2.6743]. The overall heterogeneity was Q = 4792.3756, P < 0.001. The effect size inferred by the phylogenetic meta-analysis was consistent with the results of the traditional meta-analysis, and the sigma value of the “species” in the analysis was zero, which indicated that a phylogenetic signal was not present in the calculated effects sizes. 82.4% of the species exhibited pollen limitation (i.e., a positive effect size and the confidence intervals that did not overlap zero) (Fig. 2.). These results indicated a severe pollen limitation for the flowering plants growing in the East Himalaya-Hengduan Mountains region. For the present dataset, we used a funnel plot and tested its symmetry to indicate publication bias. The p value of the symmetry test was smaller than 0.001, which indicated there might be a possibility for publication bias. Several studies used an influence plot to exclude outliers, thus making the dataset perfectly fit the symmetry test [15]. The seed productions of the hand- and open-pollination treatments were generally not the key results for most publications, and studies with different degrees of pollen limitation would not be treated differently for publication. Moreover, the Rosenberg fail-safe number is 80,721 in the present study, which is much larger than the critical value (1080); thus, it is safe to conclude that the results are robust with the consideration of publication bias [31]. Considering that a high degree of pollen limitation would most likely be the reality for flowering plants in the East Himalaya-Hengduan Mountains region [36], we did not eliminate specific values to make the data fit symmetry test.

### Pollen limitation associated with plant features, flowering time and elevation

**Fig. 3:**
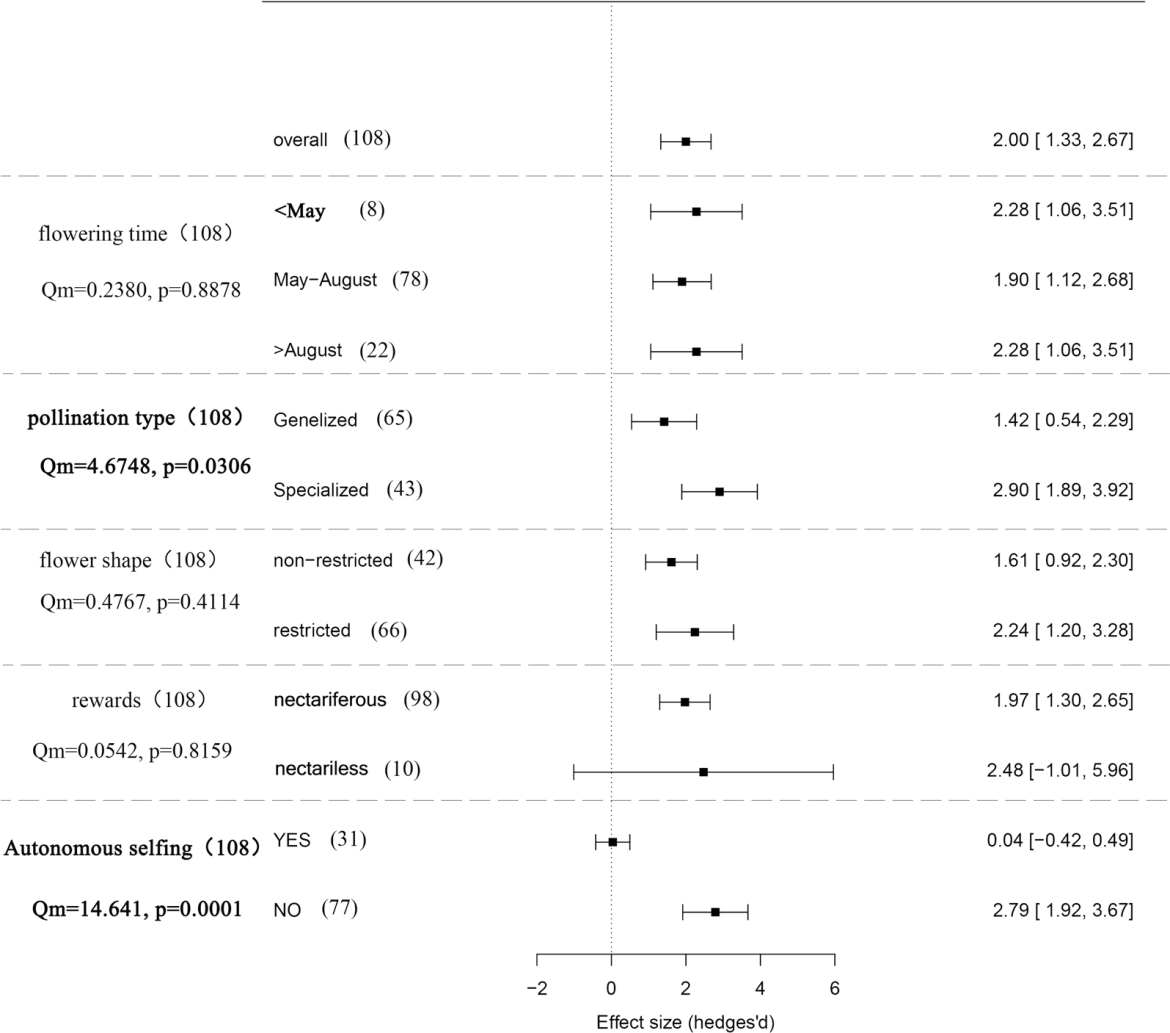
Effect size and the 95% confidence interval of the pollination limitation in the East Himalaya-Hengduan Mountains. The sample sizes of each category, the within-category heterogeneity (Q_m_) and *P* value are presented

**Fig. 4:**
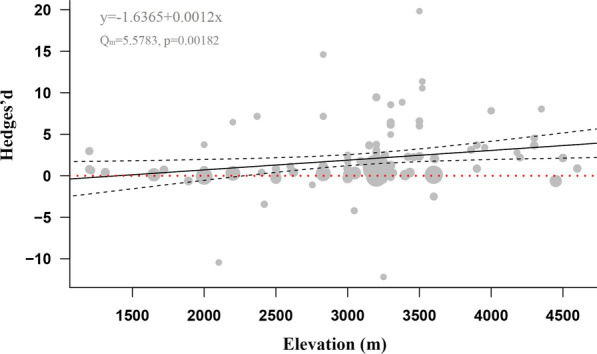
Correlation between elevation and the degree of pollination limitation. The within-category heterogeneity (Q_m_), *P* value and correlation coefficient are presented. Red dash line is the abline, the black solid line is the fitting line between elevation and the degree of pollen limitation, 2 black dash lines show the interval confidence

Five explanatory variables were included in the present analysis, and we did not find that the flower shape, reward type or flowering time accounted for the heterogeneity in the dataset significantly. Elevation, the capacity for autonomous selfing and the pollination type accounted for a significant amount of the heterogeneity in the dataset (Fig. 3). The degree of pollen limitation showed a significant correlation with elevation (Fig. 4). We did not find significant interactions between elevation and flowering time (*p* = 0.1821), between pollination type and the capacity of autoselfing (*p* = 0.2702), or between elevation and the capacity of autoselfing on the degree of pollen limitation (p = 0.1821). Only a positive interaction between pollination type and flower shape was found on the degree of pollen limitation (Qm = 4.1285, *p* = 0.0422).

## Discussion

Pollen limitation is frequently reported in various flowering plants in different places around the world [37, 38]. Severe pollen limitation could cause a decrease in resources for flowering plants [6]. The present study reveals a severe pollen limitation for flowering plants growing in East Himalaya-Hengduan Mountains region. We found that two major plant features, including the capacity for autonomous self-reproduction and the pollination pattern, accounted for a significant amount of the variation in the pollen limitation in the analysis. We also found a statistically significant relationship between elevation and the degree of pollen limitation, which indicates that pollen limitation is more frequent among the flowering plants in high elevation places compared to those growing in low elevation places in this region.

Comparing with similar studies conducted in different areas around the world (pollen limitation of alpine plants [14], flowering plants in Brazil Atlantic forest [15]; and pollen limitation in the biodiversity hotspots [13]), our results showed a much higher degree of pollen limitation in East Himalaya-Hengduan Mountains region (Hedges’ d = 2.004, but the overall hedges’d of alpine plants conducted by García-Camacho and Toland was only less than 1 [14]). According to the investigations conducted by various researchers, pollination service is generally less available in high elevation region [39]. Our result is consistent with the assumption that plants of high elevational places would more likely to be pollen limited. Thus, we assumed that our result is tough and the cause for relative low degree of pollen limitation in García-Camacho and Toland’s research might be the sample size problem (only contains 24 species). The other proper reason for severe pollen limitation in east-Himalaya and Hengduan Mountain region could also be that plant species in biodiversity hotspots suffer more from pollen limitation than those in lower diversity regions, probably because strong pollination service competition among conspecific species or significant pollinator abundance declines in such area [13].

As expected, the capacity for autonomous self-reproduction accounts for a significant amount of the heterogeneity in the dataset. Plants that are capable of autonomous self-reproduction would largely rely on themselves for sexual reproduction; thus, the reproductive success of most autonomous selfing species does not rely on external pollination services; in other words, autonomous selfing could provide reproductive assurance for most plants [40]. Self-incompatibility is reported to be an essential trait correlated with the pollen limitation as well [41], and was included in similar meta-analysis but no significant correlation with the degree of pollen limitation was shown [15]. It might be because self-incompatibility system is not the direct cause for pollination success in most cases. Due to other floral mechanisms such as herkogamy [42], dichogamy [43] and flexistyly [44], many self-compatible plants could not effectively autonomous self-reproduce as well. Therefore, we focus on the capacity for self-reproduction but not self-incompatibility system of plants in the present work. Our works show that the capacity for autonomous selfing could significantly explain that variation of the effective size. In fact, to our knowledge, many alpine plants rely on autonomous selfing for their reproduction success [45, 46], some of them even have evolved interesting delayed selfing mechanism [47].

In the present work, the relationship between the degree of pollen limitation with four major plant features (the capacity of autonomous selfing, the pollination pattern, the flower shape and the reward type) were tested. We found that the flower shape, the rewards type and the flowering time could not effectively explain the variation of the pollen limitation in our dataset. The pollination pattern, i.e., plants with generalized vs. specialized pollination, accounts for a significant amount of the heterogeneity in the dataset. This result indicates that the plants with generalized pollination could probably more effectively avoid pollen limitations in this area [48]. Besides this, a significant interaction between pollination pattern and flower shape was also found. For general, the pollination pattern and flower shape are closely correlated. The generalized pollinated flowers are in a non-restricted shape, such as the flowers of Asteraceae, Ranunculaceae and Rosaceae [49]; and the flowers in restricted shape are usually specialized pollinated by one functional group [50]. This might be the possible reason why pollination pattern and flower shape are closely interacted on the pollen limitation.

In addition, a clear correlation between elevation and the degree of pollen limitation was shown in our results. For a common view for most scientists, the richness, abundance and variability in pollinators are thought to decline from low to high elevation areas [51, 52]. Therefore, the degree of pollen limitation of the flowering plants should be severer with the increase of elevation since the abundance and variability of pollinators decrease. Several studies have investigated how elevation would affect the pollen limitation by comparing the pollination service between mountain and lowland populations [39], but limited studies have quantitatively assessed the correlation between elevation and the degree of pollen limitation. Our results provide clear evidence that pollen limitation decrease from the low to high elevation areas. Interestingly, our results show a tendency that the degree of pollen limitation increases from 1200 to 3500 m and decrease from 3500 m to above, but not statistically significant. That might be because the proportion of autonomous selfing plants that lived over 3500 m height is higher than those in other places.

The explanatory factor for the pollen limitation is not always the same in different areas for different plants [13, 15]. The capacity of autonomous self-reproduction, which is the most effective way in providing reproductive assurance for plants, is the most significant factor explaining the variation in our study. And the elevation, which would affect pollination environment in various ways, could also be an important factor affecting pollen limitation. Besides the result that flowering plants in East Himalaya-Hengduan Mountains region undergoes severe pollen limitation, the major findings of our research suggest that the capacity of autonomous selfing and elevation could be important factors affecting pollen limitation.

## Conclusion

We revealed severe pollen limitation for the flowering plants growing in East Himalaya-Hengduan Mountains, which is one of the global biodiversity hotspots. We also found that the capacity for autonomous selfing and the pollination pattern had significant effect on the pollen limitation. In addition, we found a clear relationship between elevation and the degree of pollen limitation; that is, plants grown in high elevation places suffer more severe pollen limitations. The present study is the first to synthesize an analysis of the degree of pollen limitation in this area and to quantitatively assess the relationship between elevation and the degree of pollen limitation.

## Supplementary information

**Additional file 1: Table S1.** Data, reference, location, elevation, plant features, effect size and its variance for the species included in this review.

## Data Availability

The original data from this paper are presented in the Additional file 1. Table S1.
